# Supplementary material for: Editing of the urease gene by CRISPR-Cas in the diatom Thalassiosira pseudonana
Source: Plant Methods. 2016 Nov 24;12:49. doi: 10.1186/s13007-016-0148-0 (PMC5121945; doi:10.1186/s13007-016-0148-0)
Supplement: Supplementary file 1 — Additional file 1: Figure S1. Final spreadsheet for choosing sgRNAs. [file 13007_2016_148_MOESM1_ESM.pdf]

Target sequence	bp No	Cas9 cut	GC	5' Nt	PAM	Location	Strand	Sense sequence	sgRNA score	Restriction sites
GCCTCGAGTAGAAGTCACCG	41	44	0.4	G	TGG	Intron1	-	CGGTGACTTCTACTCGAGGC	0.890	HphI
GCTCATTGCAAGGATTCAAG	915	932	0.45	G	CGG	Exon3	+	GCTCATTGCAAGGATTCAAG	0.839	
GTCGTAATCAAGTATTGCCG	158	175	0.4	G	GGG	Exon2	+	GTCGTAATCAAGTATTGCCG	0.791	HpaII
GTTGGGGTGAGATTGAAAAG	755	772	0.4	G	AGG	Exon3	+	GTTGGGGTGAGATTGAAAAG	0.790	
GACTATTCATGCTTACCACA	1659	1676	0.55	G	CGG	Exon3	+	GACTATTCATGCTTACCACA	0.773	
GCATCATTTCCACATGACCA	1380	1383	0.3	G	GGG	Exon3	-	TGGTCATGTGGAAATGATGC	0.692	
GTGACTTTGGTGAATCTTGG	676	693	0.35	G	CGG	Exon3	+	GTGACTTTGGTGAATCTTGG	0.683	
GTAGCCGATGGAGATCGTTG	739	756	0.6	G	GGG	Exon3	+	GTAGCCGATGGAGATCGTTG	0.653	
GATTTAAGTGTGTTTCGTGG	319	336	0.45	G	TGG	Exon2	+	GATTTAAGTGTGTTTCGTGG	0.648	
GGATGGGACCAAGTTGTTGA	225	242	0.45	G	CGG	Exon2	+	GGATGGGACCAAGTTGTTGA	0.600	
GAGAGGTCATCACTCGTACG	2048	2065	0.35	G	TGG	Exon4	+	GAGAGGTCATCACTCGTACG	0.563	MaeII Csp6I SplI
GGAGTCACTACAATGTTTGG	1306	1323	0.4	G	AGG	Exon3	+	GGAGTCACTACAATGTTTGG	0.534	
GGTGCATGATCCTATCGGGA	246	263	0.65	G	GGG	Exon2	+	GGTGCATGATCCTATCGGGA	0.533	
GGTAATCCGGATACAATGAA	1138	1155	0.5	G	TGG	Exon3	+	GGTAATCCGGATACAATGAA	0.502	TspDTI
GTTTCCGATCTAATGTCCAT	121	138	0.55	G	CGG	Exon2	+	GTTTCCGATCTAATGTCCAT	0.500	BccI
GAATGGATGGATCAAGGTGG	1905	1908	0.55	G	TGG	Exon4	-	CCACCTTGATCCATCCATTC	0.485	
GATTGTACCAGGTCAAGTGA	411	428	0.4	G	TGG	Intron2	+	GATTGTACCAGGTCAAGTGA	0.481	
GGAGATAGACCTATTCAAGT	529	546	0.5	G	TGG	Exon3	+	GGAGATAGACCTATTCAAGT	0.479	
GCAACTACTGATGTAATTGC	1198	1215	0.5	G	GGG	Exon3	+	GCAACTACTGATGTAATTGC	0.478	MluCI
GATGGTATCGGAGAACGATT	2467	2484	0.45	G	GGG	Exon4	+	GATGGTATCGGAGAACGATT	0.477	
GCGATAGCGTGGGCTCAGAT	2317	2334	0.5	G	GGG	Exon4	+	GCGATAGCGTGGGCTCAGAT	0.476	BccI BseMII
GTGGAGGCGACATTTCCGGA	208	225	0.4	G	TGG	Exon2	+	GTGGAGGCGACATTTCCGGA	0.465	BseGI HpaII BspMII

Figure S1
